# Supplementary material for: Anaerobic fungi in the tortoise alimentary tract illuminate early stages of host-fungal symbiosis and Neocallimastigomycota evolution
Source: Nat Commun. 2024 Mar 28;15:2714. doi: 10.1038/s41467-024-47047-4 (PMC10978972; doi:10.1038/s41467-024-47047-4)
Supplement: Supplementary file 1 — Supplementary information [file 41467_2024_47047_MOESM1_ESM.pdf]

Supplementary text for:

Anaerobic fungi in the tortoise alimentary tract illuminate early stages of host-fungal symbiosis and *Neocallimastigomycota* evolution

Carrie J. Pratt[1], Casey H. Meili[1], Adrienne L. Jones[1], Darian K. Jackson1, Emma E. England[1], Yan Wang[2], Steve Hartson[3], Janet Rogers[3], Mostafa S. Elshahed[1], and Noha H. Youssef[1*]

Supplementary results

Comparative transcriptomic analysis of tortoise- and mammalian-affiliated AGF isolates.

Comparative analysis of the 7 transcriptomes originating from tortoise AGF isolates to the 52 mammalian sourced AGF transcriptomes revealed similar distinct transcript numbers, albeit with significantly shorter average length (Figure S5a, Student t-test p-value= 0.0398), and significantly higher AT content (Figure S5a, Student t-test p-value= 0.00025). The overall GO, COG, KOG, and KEGG composition did not vary by the source of isolation (mammalian versus tortoise) (Figure S5b). However, comparative gene content analysis identified distinct transcripts that are unique to both tortoise isolates (Clusters GroupA; n=384 functional clusters), unique to one of them (i.e., present in NY36 but not NY54 or the mammalian affiliated AGF isolates transcriptomes (Clusters GroupB; n=4231 functional clusters), and vice versa (Clusters GroupC; n=3199 functional clusters), or present in mammalian affiliated AGF isolates but absent from both tortoise affiliated AGF isolates (Clusters GroupD; n=1699 functional clusters). KEGG analysis of these functional clusters revealed that 66.43-72.31% of the functions unique to both (GroupA) or either (GroupB and GroupC) of the tortoise clades were related to genetic information processing, environmental information processing and cellular processes, while only 14.09-29.93% were related to metabolism. On the other hand, clusters that were unique to the mammalian isolates (GroupD) were mainly associated with a metabolic function (53.13%) (Figure S6). Further analysis of the clusters unique to the mammalian isolates revealed that most of the metabolic functions were related to carbohydrate metabolism (49.63% of metabolic functions) (Figure S6B), which in turn were enriched in CAZymes (46.01% of carbohydrate metabolism) geared towards lignocellulose degradation (13 GH families, and 1 PL family) (Figure S6C). Interestingly, 23.35% of GroupD clusters were previously shown to be acquired by

horizontal gene transfer (1). Also, the majority of the GH families enriched in the mammalian isolate transcriptomes were previously shown to be completely (red in Figure S6C), or partly (blue in Figure S6C) acquired via HGT (1). Such pattern led us to postulate that the observed curtailed capacity for substrate degradation observed in tortoise-affiliated AGF (Figure S7) is due to their possession of a limited extracellular enzyme machinery compared to mammalian-affiliated AGF, and that such limited machinery is mostly due to the lack of widespread HGT events previously observed in mammalian AGF (1).

Limited horizontal gene transfer in tortoise-associated AGF. To quantify the contribution of HGT, or lack thereof, to shaping tortoise AGF transcriptomes, we employed the HGT detection pipeline previously utilized for mammalian AGF transcriptomes. Using this pipeline, only 35 distinct HGT events (with an average of 0.16±0.05% of transcripts in the 7 sequenced T-AGF transcriptomes) (Table 1). This value is markedly lower than the 277 events previously reported from mammalian sourced AGF transcriptomes (1). In addition to the relative paucity of HGT events, two interesting patterns emerged. First, thirty of the 35 events identified in tortoise-sourced transcriptomes as horizontally transferred were also previously reported in mammalian AGF as horizontally transferred (1), with only 5 events exclusive for the tortoise sourced transcriptomes. These shared HGT events between mammalian and tortoise AGF also share the identity of the donor, with a bacterial origin for 27/30 shared HGT events and 26 of these 27 bacterial events sharing the same donor phylum. In addition, out of the four HGT events in tortoise-sourced transcriptomes with eukaryotic origin, two shared the same donor with mammalian sourced transcriptomes. These results imply the occurrence of ancient horizontal gene transfer events that were retained post-diversification of the mammalian AGF genera. Secondly, the majority of HGT events (29/35) encoded a metabolic function contributing to

survival in the anaerobic gut. These functions included recycling reduced electron carriers via fermentation (aldehyde/alcohol dehydrogenases and d-lactate dehydrogenase for ethanol and lactate production from pyruvate), *de novo* synthesis of NAD via the bacterial pathway, the acquisition of the oxygen-sensitive ribonucleoside-triphosphate reductase class III and of squalene-hopene cyclase, catalyzing the cyclization of squalene into hopene during biosynthesis of tetrahymanol (that replaced the molecular O_2-requiring ergosterol in the cell membranes of AGF). While the tortoise AGF CAZYome was significantly curtailed (Figure 5), some of the HGT events identified in tortoise-sourced transcriptomes involved CAZyme families acquired from bacterial members of the gut (Firmicutes, Bacteroidetes, and Fibrobacteres). However, the number of HGT-acquired CAZyme genes in T-AGF was extremely minor (13 events representing an average of 10.81±4.17% of the total CAZYome in the 7 sequenced transcriptomes) compared to the massive acquisition of CAZymes by HGT previously reported in M-AGF (a total of 72 events representing 24.62-40.41% of the overall CAZyome) (1).

Tortoise-affiliated AGF secretome. To examine whether the curtailed CAZyome in tortoise isolates is part of a broader pattern of an overall curtailed secretome, we compared the predicted secretome (transcriptome predicted peptides destined to the extracellular milieu as predicted by DeepLoc) of a mammalian AGF isolate, *Orpinomyces joyonii* strain AB3, to these of the tortoise isolates B1.1, and T130A (each representing one of the AGF affiliated genera NY36, and NY54, respectively). Results showed that a smaller percentage (6.98-7.02%) of tortoise isolates predicted peptides were extracellular (using DeepLoc), as opposed to 11.49% of the predicted peptides of the mammalian isolate (Figure S9A). The mammalian isolate predicted secretome was slightly more enriched in carbohydrate metabolism (Figure S9C). Further, only 11.98-12.69% of the predicted secretome was affiliated with a CAZyme family in the tortoise affiliated

strains, as opposed to 18.28% in the mammalian sourced isolate *Orpinomyces joyonii* strain AB3 (Figure S9D), with a slightly different CAZYome composition (Figure S9E).

Supplementary Tables.

Table S1. The 11 tortoise samples studied here, along with their sampling locations. All tortoises belonged to the same family but were distributed into 8 genera and 9 species. Information on conservation status was from (2), while information on feed, geographical range and natural habitat was obtained from the US Fish and Wildlife Service website (https://www.fws.gov/).

Sample name	Samling location	Family	Genus	Speceis	Common name	Conservation Status	Feed	Geographical range in the wild	Natural habitat
Sulcata_S4_Hawk	Hawk Hill Farms	*Testudinidae*	*Centrochelys*	*C. sulcata*	African Spurred Tortoise	Endangered	foliovore	Southern edge of the Sahara desert in Africa	Savannas
Sulcata_Zoo_519	OKC Zoo								
Pancake_Zoo_522			*Malacochersus*	*M. torneieri*	Pancake Tortoise	Critically Endangered	graminvore/foliovore	East Africa	Scrub forest and arid
Ploughshare_Zoo_523			*Astrochelys*	*A. yniphora*	Ploughshare Tortoise	Critically Endangered	granivore/foliovore/ [illegible]	Madagascar	Bamboo-scrub habitat
Burmese Star_Zoo_588			*Geochelone*	*G. platynota*	Burmese Star Tortoise	Critically Endangered	graminvore/foliovore	Myanmar	Deciduous forests
Texas_Zoo_590			*Gopherus*	*G. berlandieri*	Texas Tortoise	Least Concern	graminvore/frugivore	South-Central Texas in the United States southward into the Mexican states of Coahuila, Nuevo Leon, and Tamaulipas	Semi-desert areas in Mexico, and scrub forests in humid, subtropical areas in southern Texas
Impressed_Zoo_591			*Manouria*	*M. impressa*	Impressed Tortoise	Endangered	mainly mushroom, but also grass and bamboo	Southeast Asia, mainly in Myanmar Burma, southern China, Thailand, Laos, Vietnam, Cambodia, Malaysia and Northeast India	High eleveation forest areas
Indian Star_Zoo_593			*Geochelone*	*G. elegans*	Indian Star Tortoise	Vulnerable	foliovore/frugivore	India, Pakistan and Sri Lanka	Scrub forest and arid [illegible]
Galapagos_Zoo_594			*Chelonoidis*	*C. niger*	Galapagos Tortoise	Critically Endangered	graminvore/foliovore/ frugivore	The Galápagos Islands	Island humid highlands
Galapagos_Zoo_604									
Egyptian_Zoo_595			*Testudo*	*T. kleinmanni*	Egyptian Tortoise	Critically Endangered	graminvore/foliovore	Coastal Libya and Egypt	Deserts

Table S2. List of isolates obtained from eight tortoise species, the sampling location, and the candidate genus they belong to. Isolate names in boldface have been used for transcriptomic sequencing. Isolates belonging to candidate genera NY54 and NY36 have been formally characterized and named in (5). The isolate belonging to candidate genus NY56 has been extremely hard to maintain as a viable culture for subsequent analysis.

**Tortoise species**	Location	Isolate Names	Identity
Egyptian tortoise (*Testudo kleinmanni*)	Oklahoma City Zoo	**E01**	NY54
Galápagos tortoise (*Chelonoidis niger*)	Oklahoma City Zoo	G01, **G01.1**, G01.2, G01.3, G01.4, G01.5	
Indian star tortoise (*Geochelone elegans*)	Oklahoma City Zoo	N0S0.1, N0S0.3, **N0S1.1**, N0S1.3, N0S2	
Pancake tortoise (*Malacochersus tornieri*)	Oklahoma City Zoo	P00, **P01**	
Ploughshare tortoise (*Astrochelys yniphora*)	Oklahoma City Zoo	T030A, T030A.3, T130A, **T130A.3**, T230A, T230A.3, T0395, T0397, T0399	
Burmese star tortoise (*Geochelone platynotan*)	Oklahoma City Zoo	B01.1, B01.2, B01.3, **B1.1, B1.2,** B0.3	NY36
Sulcata tortoise (*Centrochelys sulcate*)	Hawk Hill Farms	S1.1, S1.2, S1.3, S1.4, S09, S19, S29	
Texas Tortoise (*Gopherus berlandieri*)	Oklahoma City Zoo	X1	NY56

Table S3. Wilcoxon test adjusted p-values for the significance of difference in GH, CE, and PL composition for the CAZy families in red text in Figure S8.

CAZy family		Group1	Group2	Adjusted p-value	
GHs	GH10	*Anaeromycetaceae* (n=9)	Tortoise-sourced (n=7)	0.07	ns
		Caecomycetaceae (n=6)		0.13	ns
		Neocallimastigaceae 1 (n=8)		2.35E-13	****
		Neocallimastigaceae 2 (n=8)		1.46E-04	***
	GH11	*Anaeromycetaceae* (n=9)	Tortoise-sourced (n=7)	3.52E-15	****
		Caecomycetaceae (n=6)		6.24E-16	****
		Neocallimastigaceae 1 (n=8)		9.63E-43	****
		Neocallimastigaceae 2 (n=8)		9.77E-20	****
	GH13	*Anaeromycetaceae* (n=9)	Tortoise-sourced (n=7)	9.34E-05	****
		Caecomycetaceae (n=6)		0.08	ns
		Neocallimastigaceae 1 (n=8)		3.13E-06	****
		Neocallimastigaceae 2 (n=8)		1.67E-04	***
	GH16	*Anaeromycetaceae* (n=9)	Tortoise-sourced (n=7)	0.06	ns
		Caecomycetaceae (n=6)		1	ns
		Neocallimastigaceae 1 (n=8)		3.50E-06	****
		Neocallimastigaceae 2 (n=8)		0.15	ns
	GH3	*Anaeromycetaceae* (n=9)	Tortoise-sourced (n=7)	1.65E-03	**
		Caecomycetaceae (n=6)		1	ns
		Neocallimastigaceae 1 (n=8)		1.29E-17	****
		Neocallimastigaceae 2 (n=8)		2.68E-07	****
	GH43	*Anaeromycetaceae* (n=9)	Tortoise-sourced (n=7)	4.31E-20	****
		Caecomycetaceae (n=6)		1.04E-15	****
		Neocallimastigaceae 1 (n=8)		1.69E-25	****
		Neocallimastigaceae 2 (n=8)		1.53E-19	****
	GH45	*Anaeromycetaceae* (n=9)	Tortoise-sourced (n=7)	9.85E-03	**
		Caecomycetaceae (n=6)		2.70E-03	**
		Neocallimastigaceae 1 (n=8)		4.93E-06	****
		Neocallimastigaceae 2 (n=8)		9.67E-03	**
	GH48	*Anaeromycetaceae* (n=9)	Tortoise-sourced (n=7)	1	ns
		Caecomycetaceae (n=6)		1	ns
		Neocallimastigaceae 1 (n=8)		5.84E-03	**
		Neocallimastigaceae 2 (n=8)		0.51	ns
	GH5	*Anaeromycetaceae* (n=9)	Tortoise-sourced (n=7)	0.37	ns
		Caecomycetaceae (n=6)		1	ns
		Neocallimastigaceae 1 (n=8)		6.65E-20	****
		Neocallimastigaceae 2 (n=8)		1.37E-04	***
	GH6	*Anaeromycetaceae* (n=9)	Tortoise-sourced (n=7)	1.70E-06	****
		Caecomycetaceae (n=6)		2.31E-05	****
		Neocallimastigaceae 1 (n=8)		2.24E-19	****
		Neocallimastigaceae 2 (n=8)		4.07E-14	****
	GH9	*Anaeromycetaceae* (n=9)	Tortoise-sourced (n=7)	2.67E-04	***
		Caecomycetaceae (n=6)		1	ns
		Neocallimastigaceae 1 (n=8)		5.10E-04	***
		Neocallimastigaceae 2 (n=8)		2.07E-02	*
CEs	CE1	*Anaeromycetaceae* (n=9)	Tortoise-sourced (n=7)	4.26E-21	****
		Caecomycetaceae (n=6)		1.02E-11	****
		Neocallimastigaceae 1 (n=8)		2.57E-12	****
		Neocallimastigaceae 2 (n=8)		2.11E-06	****
	CE2	*Anaeromycetaceae* (n=9)	Tortoise-sourced (n=7)	0.15	ns
		Caecomycetaceae (n=6)		4.56E-05	****
		Neocallimastigaceae 1 (n=8)		1.11E-02	*
		Neocallimastigaceae 2 (n=8)		0.47	ns
	CE3	*Anaeromycetaceae* (n=9)	Tortoise-sourced (n=7)	4.46E-02	*
		Caecomycetaceae (n=6)		1.81E-03	**
		Neocallimastigaceae 1 (n=8)		0.33	ns
		Neocallimastigaceae 2 (n=8)		Not compared[$]	
	CE4	*Anaeromycetaceae* (n=9)	Tortoise-sourced (n=7)	0.08	ns
		Caecomycetaceae (n=6)		3.93E-02	*
		Neocallimastigaceae 1 (n=8)		7.06E-14	****
		Neocallimastigaceae 2 (n=8)		7.15E-07	****
	CE6	*Anaeromycetaceae* (n=9)	Tortoise-sourced (n=7)	1.34E-05	****
		Caecomycetaceae (n=6)		1.14E-03	**
		Neocallimastigaceae 1 (n=8)		1.55E-10	****
		Neocallimastigaceae 2 (n=8)		7.27E-06	****
PLs	PL1	*Anaeromycetaceae* (n=9)	Tortoise-sourced (n=7)	1	ns
		Caecomycetaceae (n=6)		1	ns
		Neocallimastigaceae 1 (n=8)		2.04E-12	****
		Neocallimastigaceae 2 (n=8)		2.56E-05	****
	PL3	*Anaeromycetaceae* (n=9)	Tortoise-sourced (n=7)	0.44	ns
		Caecomycetaceae (n=6)		1	ns
		Neocallimastigaceae 1 (n=8)		8.18E-07	****
		Neocallimastigaceae 2 (n=8)		0.24	ns

Table S4. BUSCO output for transcriptome percent completeness. BUSCO (3) was used to assess transcriptome completeness using the fungi_odb10 dataset (modified to remove 155 mitochondrial protein families as previously suggested (4).

BUSCO output	*Testudinimyces gracilis*					*Astrotestudinimyces divisus*	
	BO1	EO1	GO1	NOS1	T130A	B1.1	B1.2
Complete BUSCOs	438	312	510	591	342	298	370
Fragmented BUSCOs	111	64	89	2	120	155	130
Missing BUSCOs	54	127	4	10	141	150	103
% Completeness	91.04	78.94	99.34	0.98	76.62	75.12	82.92

Table S5. Orbitrap Fusion Method Summary

Global Settings

- Use Static Source Gasses
- Use Ion Source Settings from Tune = Not Checked
- Method Duration (min)= 78
- Ion Source Type = NSI
- Spray Voltage = Static
- Spray Voltage: Positive Ion (V) = 1900
- Spray Voltage: Negative Ion (V) = 600
- Gas Mode = Static
- Infusion Mode (LC)= False
- Sweep Gas (Arb) = 0
- Ion Transfer Tube Temp (°C) = 300
- APPI Lamp = Not in use
- FAIMS Mode = Not Installed
- Application Mode = Peptide
- Pressure Mode = Standard
- Default Charge State = 2
- Advanced Peak Determination = True

Experiment 1

- Experiment Name = Universal Method
- Start Time (min) = 16
- End Time (min) = 78
- Cycle Time (sec) = 5
  - Scan MasterScan
    - Desired minimum points across the peak = 6
    - MSn Level = 1
    - Use Wide Quad Isolation = True
    - Detector Type = Orbitrap
    - Orbitrap Resolution = 120K
    - Mass Range = Normal
    - Scan Range (m/z) = 375-1575
    - Maximum Injection Time (ms) = 50
    - AGC Target = 500000
    - Normalized AGC Target = 125%
    - Microscans = 1
    - Maximum Injection Time Type = Custom
    - RF Lens (%) = 60
    - Use ETD Internal Calibration = False
    - DataType = Centroid
    - Polarity = Positive
    - Source Fragmentation = False
    - Scan Description =
    - Enhanced Resolution Mode = Off
  - Filter MIPS
    - Relax Restrictions when too few Precursors are Found = True
    - MIPS Mode = Peptide
  - Filter ChargeState
    - Include charge state(s) = 2-6
    - Include undetermined charge states = False

Filter DynamicExclusion

Exclude after n times = 1

Exclusion duration (s) = 45

Mass Tolerance = ppm

Mass tolerance low = 10

Mass tolerance high = 10

Use Common Settings = False

Exclude isotopes = True

Perform dependent scan on single charge state per precursor only = False

Data Dependent Properties

Data Dependent Mode= Cycle Time

Scan Event 1

Scan ddMSnScan

Desired minimum points across the peak = 6

MSn Level = 2

Isolation Mode = Quadrupole

Enable Intelligent Product Acquisition for MS2 Isolation = False

Isolation Window = 0.8

Isolation Offset = Off

Reported Mass = Original Mass

Multi-notch Isolation = False

Scan Range Mode = Auto

Scan Priority= 1

Collision Energy Mode = Fixed

ActivationType = HCD

Collision Energy (%) = 32

Detector Type = IonTrap

Ion Trap Scan Rate = Rapid

Maximum Injection Time (ms) = 35

AGC Target = 10000

Inject ions for all available parallelizable time = False

Normalized AGC Target = 100%

Microscans = 1

Maximum Injection Time Type = Dynamic

Use ETD Internal Calibration = False

DataType = Centroid

Polarity = Positive

Source Fragmentation = False

Scan Description =

Time Mode = Unscheduled

Enhanced Resolution Mode = Off

Supplementary Figures.

Figure S1. Maximum likelihood phylogenetic tree in Figure 1D with the wedges of the three tortoise affiliated genera expanded and including sequences from the current culture-independent study. All other genera are shown as collapsed wedges and names are color coded by genus as shown in the figure legend.

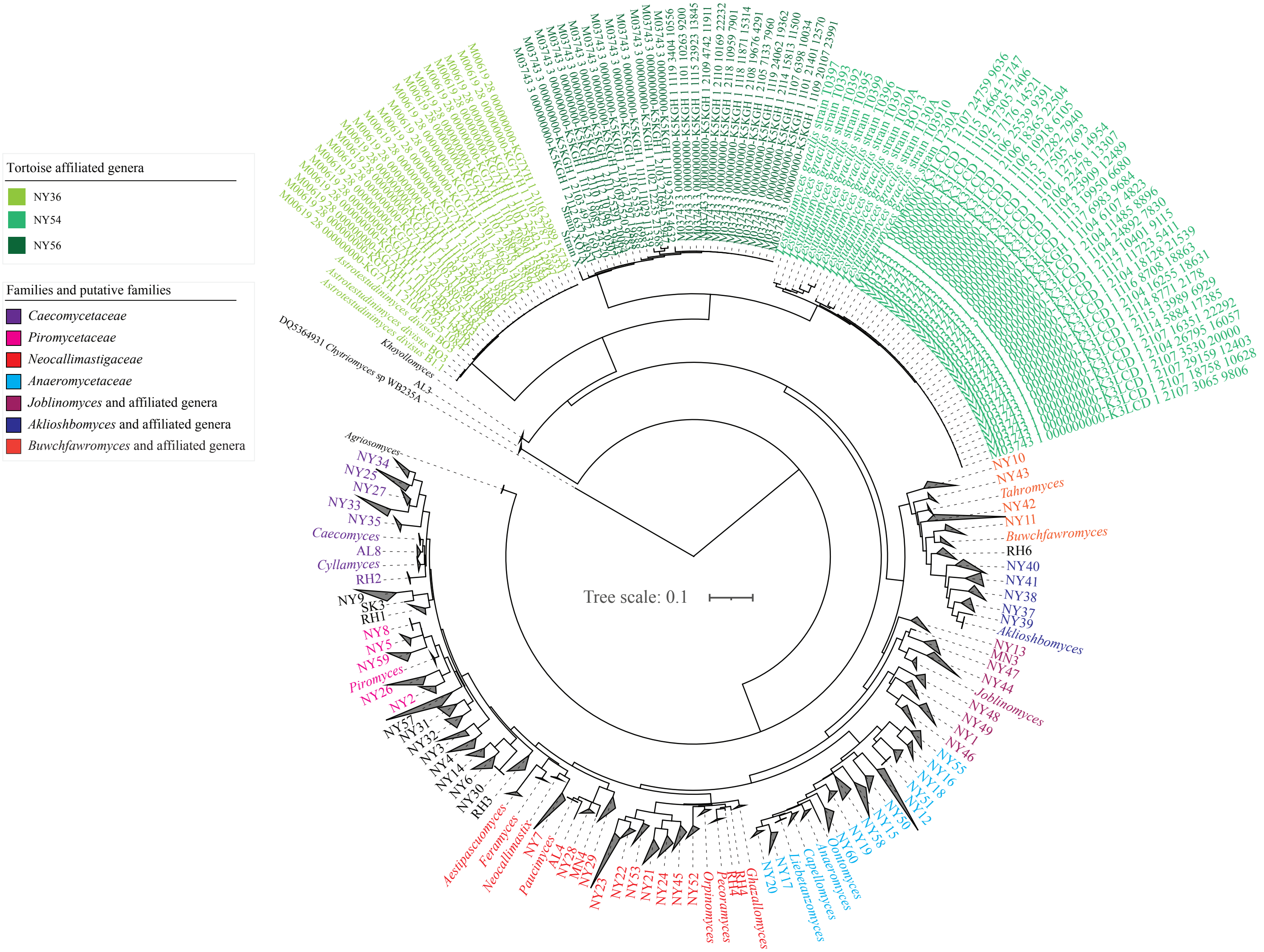

Tortoise affiliated genera
NY36
NY54
NY56
Families and putative families
Caecomycetaceae
Piromycetaceae
Neocallimastigaceae
Anaeromycetaceae
Joblinomyces and affiliated genera
Aklioshbomyces and affiliated genera
Buwchfawromyces and affiliated genera
Tree scale: 0.1
Testudinimyces gracilis
Astrotestudinimyces divisus
Khoyollomyces
AL3
DQ536493 Chytriomyces sp WB235A
Agriosomyces
NY34
NY25
NY27
NY33
NY35
Caecomyces
AL8
Cyllamyces
RH2
NY9
SK3
RH1
NY8
NY5
NY59
Piromyces
NY26
NY2
NY57
NY31
NY32
NY3
NY4
NY14
NY6
NY30
RH3
Aestipascuomyces
Feramyces
Neocallimastix
NY7
Paucimyces
AL4
NY28
MN4
NY29
NY23
NY22
NY53
NY21
NY24
NY45
NY52
Orpinomyces
Pecoramyces
RH4
Ghazallomyces
NY20
NY17
Liebetanzomyces
Capellomyces
Anaeromyces
Oontomyces
NY60
NY19
NY58
NY15
NY50
NY12
NY51
NY18
NY16
NY55
NY46
NY1
NY49
NY48
Joblinomyces
NY44
NY47
MN3
NY13
Aklioshbomyces
NY39
NY37
NY38
NY41
NY40
RH6
Buwchfawromyces
NY11
NY42
Tahromyces
NY43
NY10

Figure S2. Patterns of AGF alpha and beta diversity in the 11 tortoise samples studied in comparison to a subset of mammalian zoo-sourced hosts previously studied (2) (Dataset 1B). (A) Box and whisker plots showing the distribution of 4 alpha diversity measures (observed number of genera (Sobs), Shannon, Simpson, and Inverse Simpson) for the mammalian versus reptilian zoo-housed animals. Results of Wilcoxon signed rank test for pairwise comparison of tortoise (pink) alpha diversity indices to mammals (cyan; n=11) are shown above the boxplots. (B) Principal coordinate analysis (PCoA) plot based on the phylogenetic similarity-based index weighted Unifrac. The percentage variance explained by the first two axes are displayed on the axes, and ellipses encompassing 95% of variance are displayed. Samples and ellipses are colorcoded by host class. The table on the left is showing results from PERMANOVA analysis for the significance of host class in explaining sample variances. Adonis p-value and R^2 are shown. Source data are provided as a Source Data file.

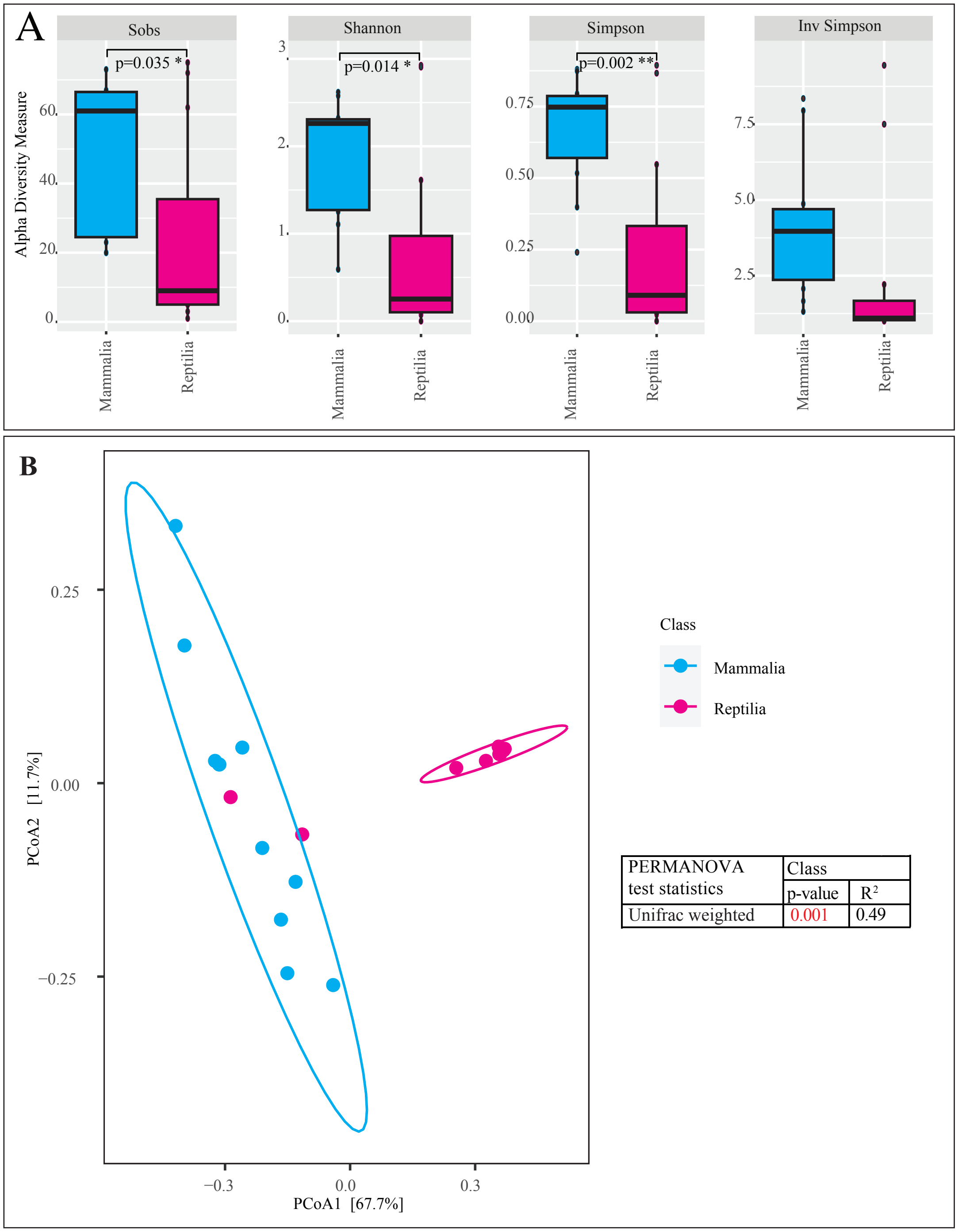
A
Sobs
Shannon
Simpson
Inv Simpson
Alpha Diversity Measure
p=0.035 *
p=0.014 *
p=0.002 **
Mammalia
Reptilia
B
Class
Mammalia
Reptilia
PCoA2 [11.7%]
PCoA1 [67.7%]
PERMANOVA test statistics
Class
p-value
R²
Unifrac weighted
0.001
0.49

Figure S3. Isolates belonging to the three putative genera growing in liquid RFC media. Isolate names and genus are indicated on the right of each tube.

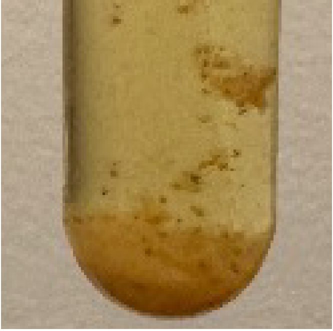

NY54

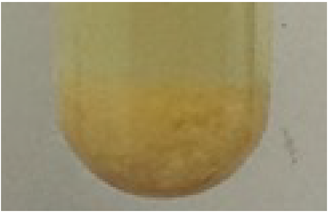

NY36

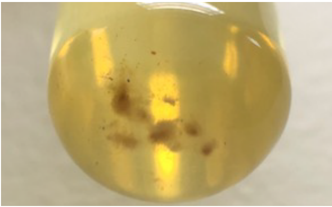

NY56

Figure S4. Phylogenomic tree of *Neocallimastigomycota* based on 670 genome-wide markers as previously described in (6). The tree was constructed using the 8 transcriptomic datasets generated in this study, as well as 52 transcriptomic datasets from 14 AGF genera previously utilized (6). In addition, 5 outgroup *Chytridiomycota* genomes (*Chytriomyces* sp. strain MP 71, *Entophlyctis helioformis* JEL805, *Gaertneriomyces semiglobifer* Barr 43, *Gonapodya prolifera* JEL478, and *Rhizoclosmatium globosum* JEL800) were included. The tree was constructed using the maximum likelihood approach implemented in the IQ-TREE package. The numbers at the nodes represent ultrafast bootstrap values. The scale bar at the bottom indicates the number of substitutions per site in the analysis.

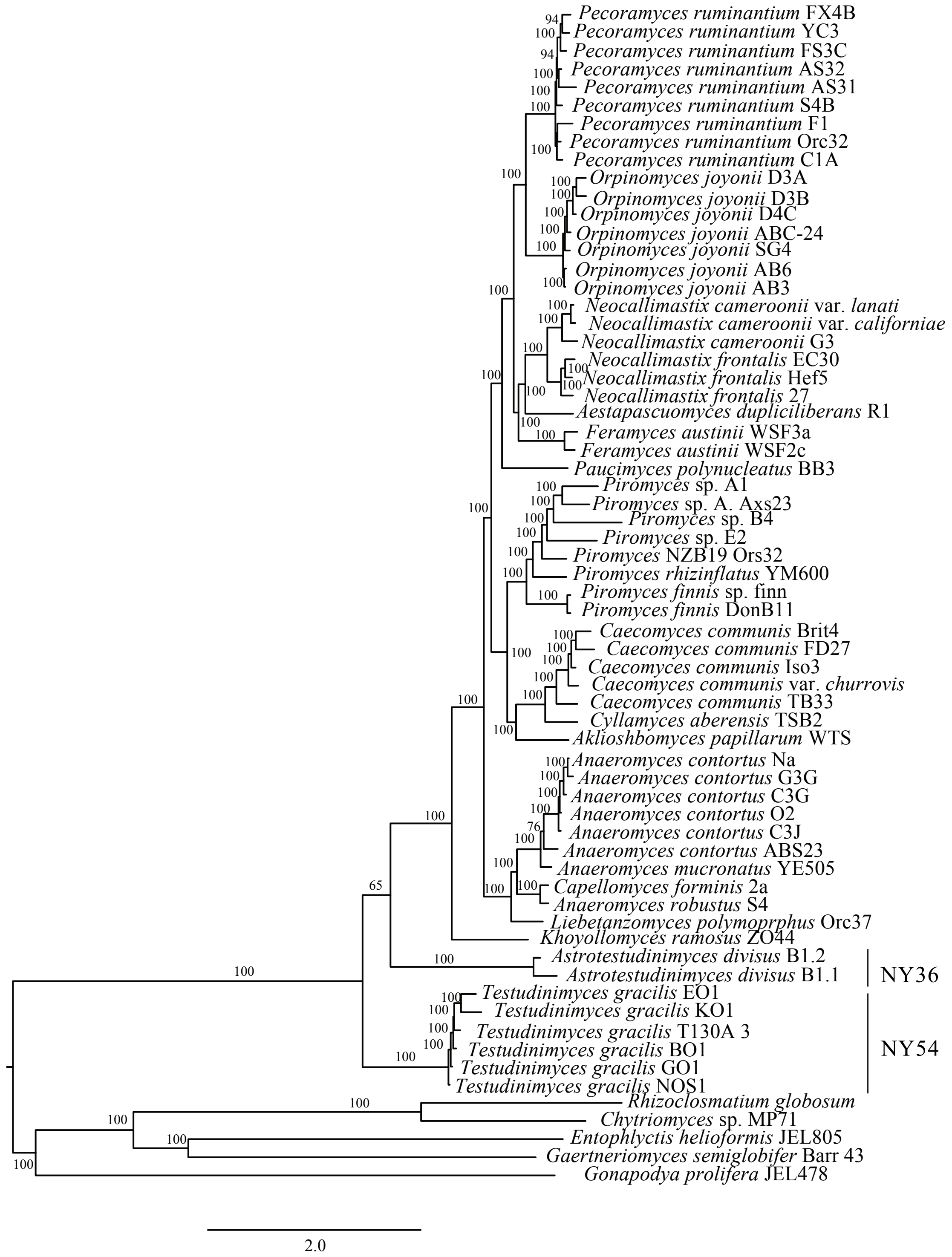

Pecoramyces ruminantium FX4B
Pecoramyces ruminantium YC3
Pecoramyces ruminantium FS3C
Pecoramyces ruminantium AS32
Pecoramyces ruminantium AS31
Pecoramyces ruminantium S4B
Pecoramyces ruminantium F1
Pecoramyces ruminantium Orc32
Pecoramyces ruminantium C1A
Orpinomyces joyonii D3A
Orpinomyces joyonii D3B
Orpinomyces joyonii D4C
Orpinomyces joyonii ABC-24
Orpinomyces joyonii SG4
Orpinomyces joyonii AB6
Orpinomyces joyonii AB3
Neocallimastix cameroonii var. lanati
Neocallimastix cameroonii var. californiae
Neocallimastix cameroonii G3
Neocallimastix frontalis EC30
Neocallimastix frontalis Hef5
Neocallimastix frontalis 27
Aestapascuomyces dupliciliberans R1
Feramyces austinii WSF3a
Feramyces austinii WSF2c
Paucimyces polynucleatus BB3
Piromyces sp. A1
Piromyces sp. A. Axs23
Piromyces sp. B4
Piromyces sp. E2
Piromyces NZB19 Ors32
Piromyces rhizinflatus YM600
Piromyces finnis sp. finn
Piromyces finnis DonB11
Caecomyces communis Brit4
Caecomyces communis FD27
Caecomyces communis Iso3
Caecomyces communis var. churrovis
Caecomyces communis TB33
Cyllamyces aberensis TSB2
Aklioshbomyces papillarum WTS
Anaeromyces contortus Na
Anaeromyces contortus G3G
Anaeromyces contortus C3G
Anaeromyces contortus O2
Anaeromyces contortus C3J
Anaeromyces contortus ABS23
Anaeromyces mucronatus YE505
Capellomyces forminis 2a
Anaeromyces robustus S4
Liebetanzomyces polymoprphus Orc37
Khoyollomyces ramosus ZO44
Astrotestudinimyces divisus B1.2
Astrotestudinimyces divisus B1.1
NY36
Testudinimyces gracilis EO1
Testudinimyces gracilis KO1
Testudinimyces gracilis T130A 3
Testudinimyces gracilis BO1
Testudinimyces gracilis GO1
Testudinimyces gracilis NOS1
NY54
Rhizoclosmatium globosum
Chytriomyces sp. MP71
Entophlyctis helioformis JEL805
Gaertneriomyces semiglobifer Barr 43
Gonapodya prolifera JEL478
2.0

Figure S5. Comparative general features and gene content analysis of the 7 tortoise sourced transcriptomes generated in this study (pink), versus the 52 mammalian sourced transcriptomes generated previously (cyan) (1, 4, 7-12). (A) Distribution of transcript length (left) and GC content (right). Results of two-tailed ANOVA for pairwise comparison are shown on top. (B-C) Gene content comparison between mammalian sourced (left stacked columns) and tortoise sourced (right stacked columns) transcriptomes using GO (B), COG/KOG (C), and KEGG (D) classification. KEGG classification is further broken down into the four main categories: Metabolism, Genetic Information Processing, Environmental Information Processing, and Cellular Processes. Source data are provided as a Source Data file.

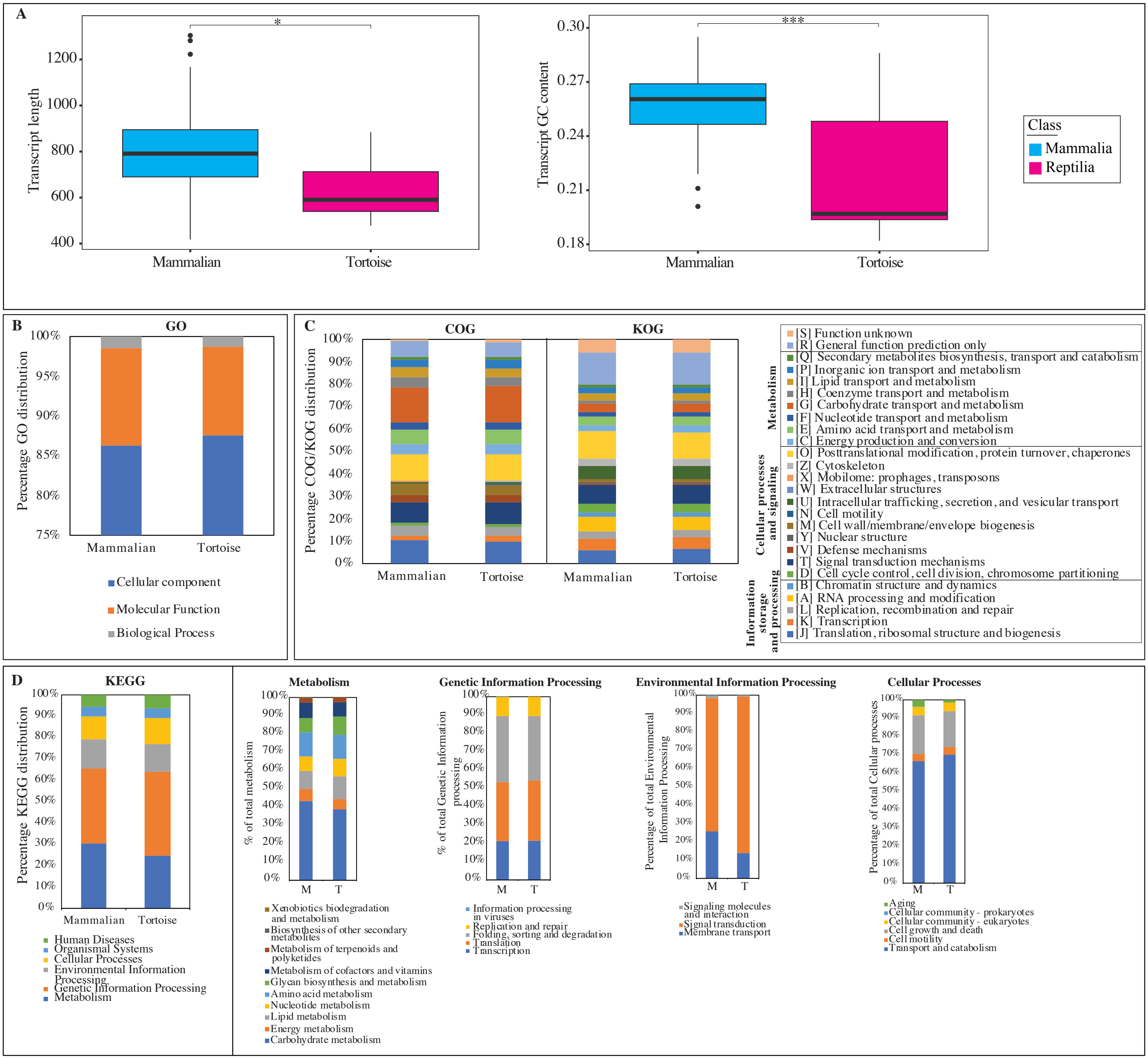
A
Transcript length
Mammalian
Tortoise
Transcript GC content
Class
Mammalia
Reptilia
B
GO
Percentage GO distribution
Cellular component
Molecular Function
Biological Process
C
COG
KOG
Percentage COG/KOG distribution
[S] Function unknown
[R] General function prediction only
[Q] Secondary metabolites biosynthesis, transport and catabolism
[P] Inorganic ion transport and metabolism
[I] Lipid transport and metabolism
[H] Coenzyme transport and metabolism
[G] Carbohydrate transport and metabolism
[F] Nucleotide transport and metabolism
[E] Amino acid transport and metabolism
[C] Energy production and conversion
[O] Posttranslational modification, protein turnover, chaperones
[Z] Cytoskeleton
[X] Mobilome: prophages, transposons
[W] Extracellular structures
[U] Intracellular trafficking, secretion, and vesicular transport
[N] Cell motility
[M] Cell wall/membrane/envelope biogenesis
[Y] Nuclear structure
[V] Defense mechanisms
[T] Signal transduction mechanisms
[D] Cell cycle control, cell division, chromosome partitioning
[B] Chromatin structure and dynamics
[A] RNA processing and modification
[L] Replication, recombination and repair
[K] Transcription
[J] Translation, ribosomal structure and biogenesis
Metabolism
Cellular processes and signaling
Information storage and processing
D
KEGG
Percentage KEGG distribution
Human Diseases
Organismal Systems
Cellular Processes
Environmental Information Processing
Genetic Information Processing
Metabolism
Metabolism
% of total metabolism
Xenobiotics biodegradation and metabolism
Biosynthesis of other secondary metabolites
Metabolism of terpenoids and polyketides
Metabolism of cofactors and vitamins
Glycan biosynthesis and metabolism
Amino acid metabolism
Nucleotide metabolism
Lipid metabolism
Energy metabolism
Carbohydrate metabolism
Genetic Information Processing
% of total Genetic Information processing
Information processing in viruses
Replication and repair
Folding, sorting and degradation
Translation
Transcription
Environmental Information Processing
Percentage of total Environmental Information Processing
Signaling molecules and interaction
Signal transduction
Membrane transport
Cellular Processes
Percentage of total Cellular processes
Aging
Cellular community - prokaryotes
Cellular community - eukaryotes
Cell growth and death
Cell motility
Transport and catabolism

Figure S6. Functional classification of MCL obtained clusters. Four groups of clusters are compared: GroupA: distinct transcripts that are present in both tortoise isolates but absent from mammalian affiliated AGF isolates, n=384 functional clusters; GroupB: distinct transcripts that are present in NY36 but not NY54 or the mammalian affiliated AGF isolates transcriptomes, n=4231 functional clusters; GroupC: distinct transcripts that are present in NY54 but not NY36 or the mammalian affiliated AGF isolates transcriptomes, n=3199 functional clusters; GroupD: distinct transcripts that are present in mammalian affiliated AGF isolates but absent from both tortoise affiliated AGF isolates, n=1699 functional clusters. (A) KEGG classification of clusters in the 4 groups. (B) Zoom in on clusters assigned a KEGG metabolism function for each of the four groups of clusters in A. (C) CAZyome classification of clusters assigned a KEGG carbohydrate metabolism in GroupD clusters. CAZy families previously shown to be completely acquired via HGT are in red text, while families previously shown to be partly acquired via HGT are in blue text (1). Source data are provided as a Source Data file.

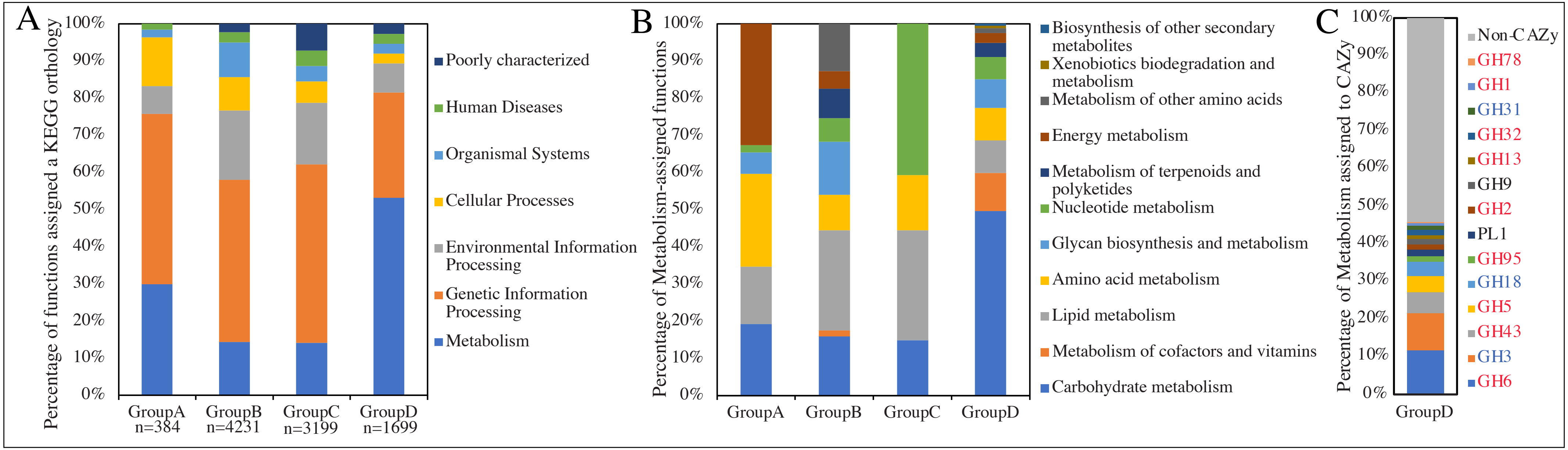
A
Percentage of functions assigned a KEGG orthology
100%
90%
80%
70%
60%
50%
40%
30%
20%
10%
0%
GroupA n=384
GroupB n=4231
GroupC n=3199
GroupD n=1699
Poorly characterized
Human Diseases
Organismal Systems
Cellular Processes
Environmental Information Processing
Genetic Information Processing
Metabolism
B
Percentage of Metabolism-assigned functions
GroupA
GroupB
GroupC
GroupD
Biosynthesis of other secondary metabolites
Xenobiotics biodegradation and metabolism
Metabolism of other amino acids
Energy metabolism
Metabolism of terpenoids and polyketides
Nucleotide metabolism
Glycan biosynthesis and metabolism
Amino acid metabolism
Lipid metabolism
Metabolism of cofactors and vitamins
Carbohydrate metabolism
C
Percentage of Metabolism assigned to CAZy
GroupD
Non-CAZy
GH78
GH1
GH31
GH32
GH13
GH9
GH2
PL1
GH95
GH18
GH5
GH43
GH3
GH6

Figure S7. Substrate utilization preferences of two representative isolates of each of the tortoise affiliated genera (NY36 strain B1.1, and NY54 strain T130A) in comparison to an *Orpinomyces joyonii* strain isolated from an American bison (strain AB3). Average gas pressure in PSI (as proxy for growth) from 4 independent growth experiments is shown on the Y-axis, while the carbon source used for growth is shown on the X-axis. Source data are provided as a Source Data file.

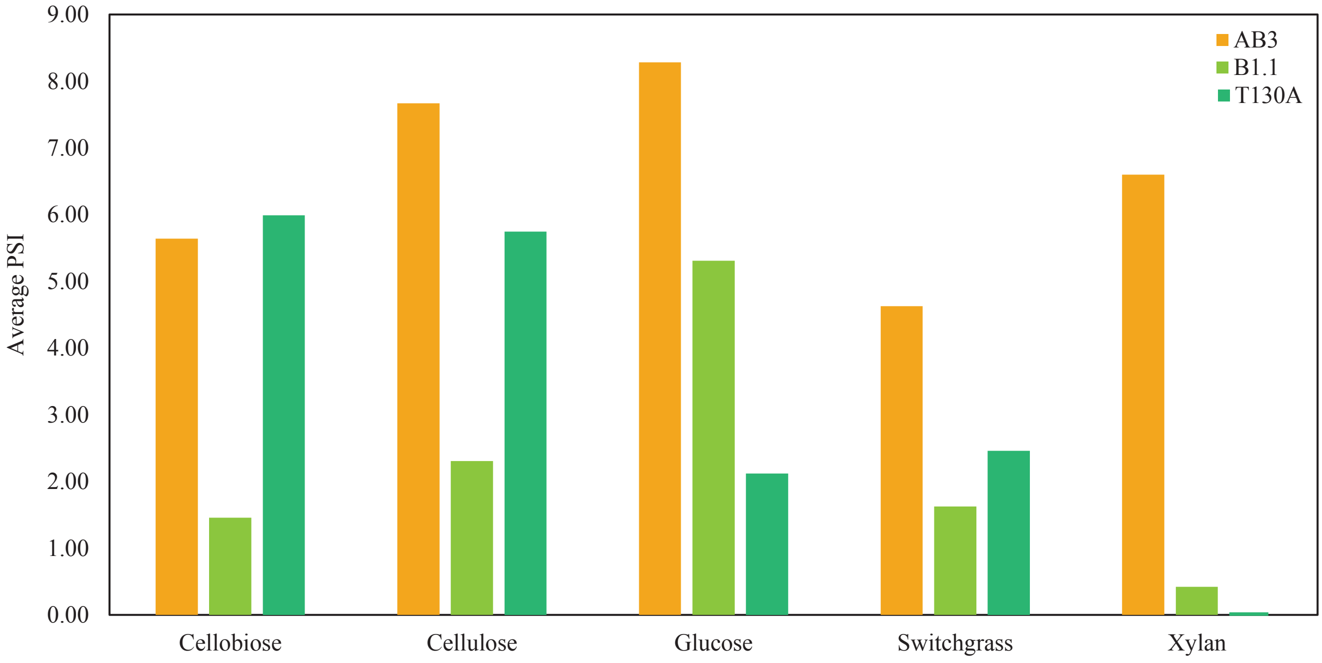

9.00
8.00
7.00
6.00
5.00
4.00
3.00
2.00
1.00
0.00
Average PSI
AB3
B1.1
T130A
Cellobiose
Cellulose
Glucose
Switchgrass
Xylan

Figure S8. CAZyome composition difference between tortoise-sourced (n=7) and four different subsets of mammalian-sourced strains belonging to the Neocallimastigomycota families *Anaeromycetaceae* (genera *Capellomyces* and *Anaeromyces*, n=9), *Caecomycetaceae* (genera *Caecomyces* and *Cyllamyces*, n=6), *Neocallimastigaceae* group 1 (genera *Orpinomyces* and *Pecoramyces*, n=8), and *Neocallimastigaceae* group 2 (genera *Neocallimastix* and *Feramyces*, n=8). Box and whisker plots for the distribution of the total number of GHs (A), CEs (B), and PLs (C) identified in the transcriptomes color coded as shown in the figure legend. Only CAZy families with >100 total hits in the entire dataset are shown, and CAZy families that were significantly more abundant in mammalian versus tortoise transcriptomes are shown in red text. Wilcoxon test adjusted p-values for the significance of difference in CAZyome composition for the CAZy families in red text are shown in Table S5. Source data are provided as a Source Data file.

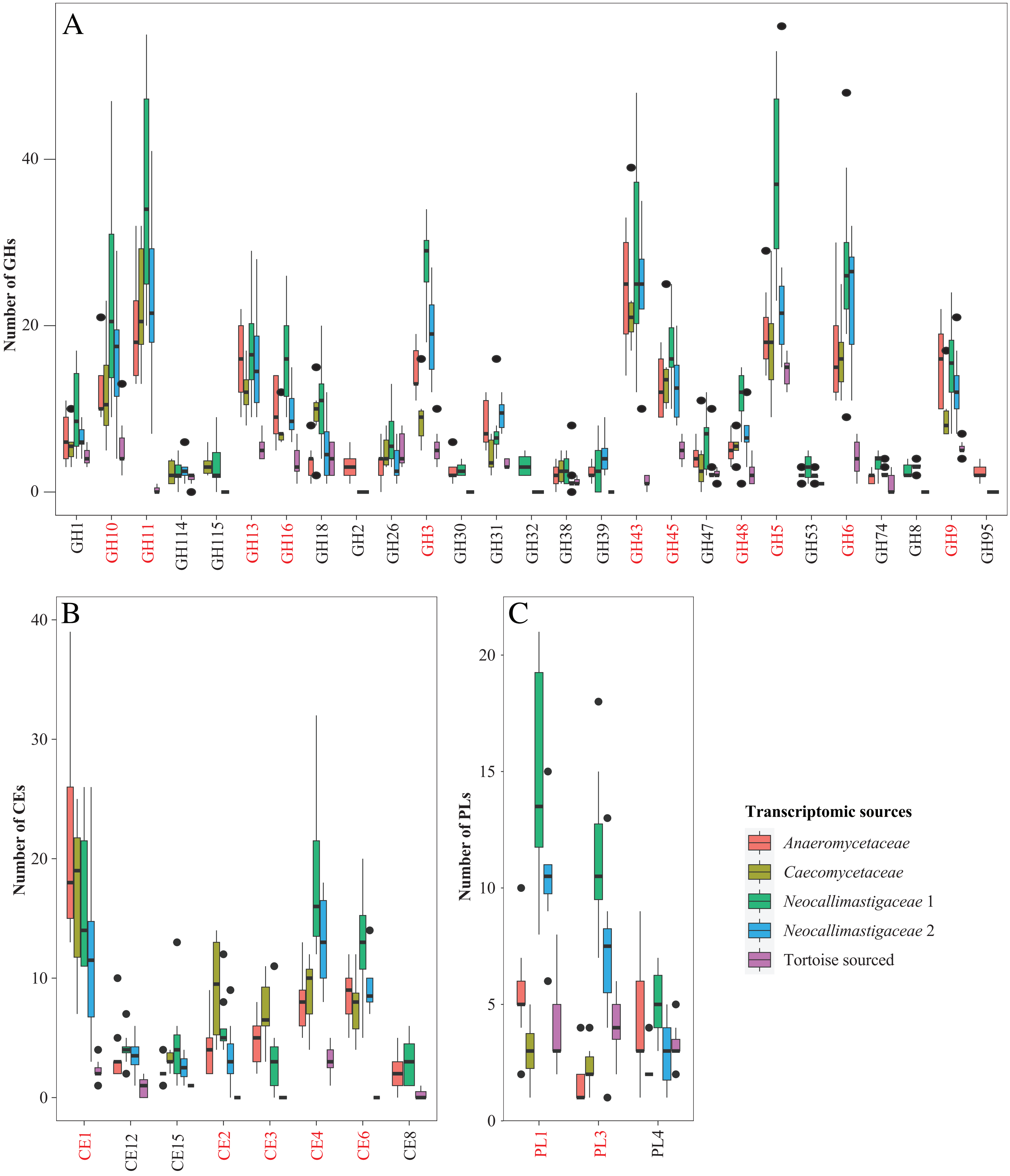

A
Number of GHs
0
20
40
GH1
GH10
GH11
GH114
GH115
GH13
GH16
GH18
GH2
GH26
GH3
GH30
GH31
GH32
GH38
GH39
GH43
GH45
GH47
GH48
GH5
GH53
GH6
GH74
GH8
GH9
GH95
B
Number of CEs
0
10
20
30
40
CE1
CE12
CE15
CE2
CE3
CE4
CE6
CE8
C
Number of PLs
0
5
10
15
20
PL1
PL3
PL4
Transcriptomic sources
Anaeromycetaceae
Caecomycetaceae
Neocallimastigaceae 1
Neocallimastigaceae 2
Tortoise sourced

Figure S9. Comparative secretome analysis. The predicted secretome (transcriptome predicted peptides destined to the extracellular milieu as predicted by DeepLoc) of a mammalian AGF isolate, *Orpinomyces joyonii* strain AB3, to these of the tortoise isolates B1.1, and T130A (each representing one of the AGF affiliated genera NY36, and NY54, respectively). (A) Predicted secretome as a percentage of total predicted peptides. (B) Functional classification of the predicted secretome in the three strains. (C) Zoom in on the predicted secretome in the three strains assigned a KEGG metabolism function. (D) Percentage of the predicted secretome in each strain with a CAZyme family prediction. (E) CAZyome composition of of the predicted secretome in each strain. All CAZYme families making up <3% of the total secretome CAZyome are grouped in “others” category. Source data are provided as a Source Data file.

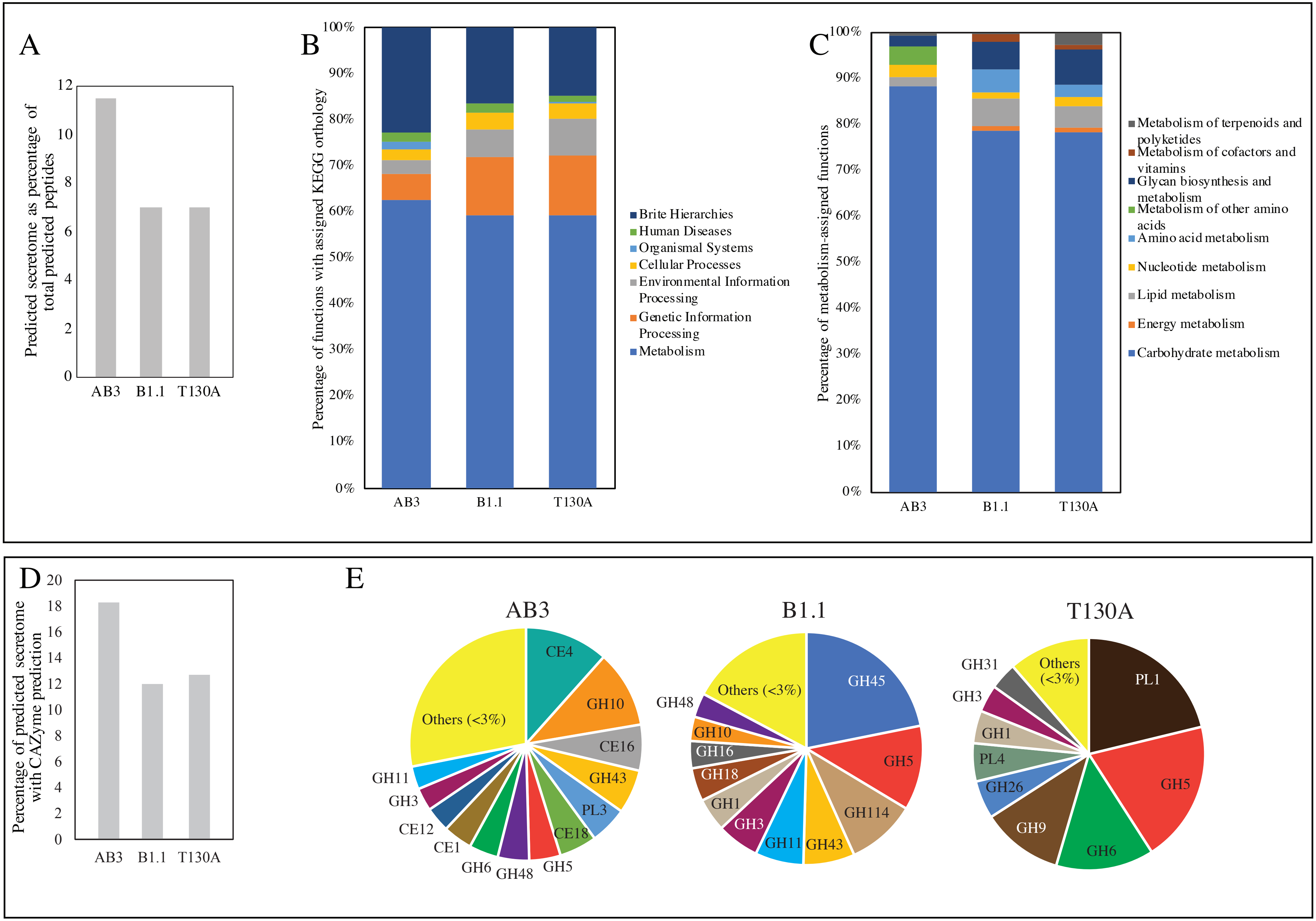
A
Predicted secretome as percentage of total predicted peptides
0
2
4
6
8
10
12
AB3
B1.1
T130A
B
Percentage of functions with assigned KEGG orthology
0%
10%
20%
30%
40%
50%
60%
70%
80%
90%
100%
Brite Hierarchies
Human Diseases
Organismal Systems
Cellular Processes
Environmental Information Processing
Genetic Information Processing
Metabolism
C
Percentage of metabolism-assigned functions
Metabolism of terpenoids and polyketides
Metabolism of cofactors and vitamins
Glycan biosynthesis and metabolism
Metabolism of other amino acids
Amino acid metabolism
Nucleotide metabolism
Lipid metabolism
Energy metabolism
Carbohydrate metabolism
D
Percentage of predicted secretome with CAZyme prediction
0
2
4
6
8
10
12
14
16
18
20
E
AB3
CE4
GH10
CE16
GH43
PL3
CE18
GH5
GH48
GH6
CE1
CE12
GH3
GH11
Others (<3%)
B1.1
GH45
GH5
GH114
GH43
GH11
GH3
GH1
GH18
GH16
GH10
GH48
Others (<3%)
T130A
PL1
GH5
GH6
GH9
GH26
PL4
GH1
GH3
GH31
Others (<3%)

Figure S10. Number of peptides predicted to be cellulosomal in the two tortoise affiliated strains. The blue bars depict the total number of peptides predicted to be cellulosomal from the transcriptomic analysis, the orange bars depict the number of cellulosomal proteins identified in the MS dataset (with the percentage of total proteins shown in top), the grey bars depict the number of proteins found to be with higher abundance in the cellulose bound fraction (ratio of cellulose-bound: biomass intensity >1), and the yellow bars depict the number of peptides found to be enriched in the cellulose bound fraction (ratio of cellulose-bound: biomass intensity >5). Source data are provided as a Source Data file.

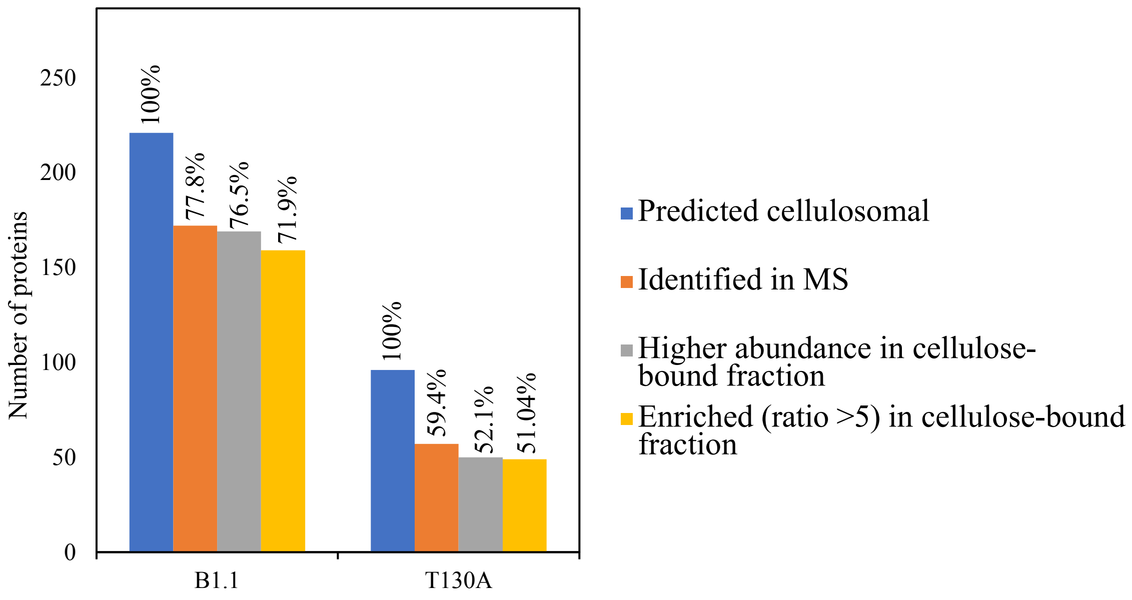

250
200
150
100
50
0
Number of proteins
100%
77.8%
76.5%
71.9%
100%
59.4%
52.1%
51.04%
B1.1
T130A
Predicted cellulosomal
Identified in MS
Higher abundance in cellulose-bound fraction
Enriched (ratio >5) in cellulose-bound fraction

References

1. C. L. Murphy *et al.*, Horizontal gene transfer forged the evolution of anaerobic gut fungi into a phylogenetically distinct gut-dwelling fungal lineage. . *Appl Environ Microbiol* **85**, e00988-00919 (2019).
2. A. G. J. Rhodin *et al.*, Global conservation status of turtles and tortoises (Order *Testudines*). *Chelonian Conserv Biol* **17**, 135-161 (2018).
3. M. Manni, M. R. Berkeley, M. Seppey, F. A. Simão, E. M. Zdobnov, BUSCO update: novel and streamlined workflows along with broader and deeper phylogenetic coverage for scoring of eukaryotic, prokaryotic, and viral genomes. *Mol Biol Evol* **38**, 4647-4654 (2021).
4. R. J. Gruninger *et al.*, Application of transcriptomics to compare the carbohydrate active enzymes that are expressed by diverse genera of anaerobic fungi to degrade plant cell wall carbohydrates. *Front Microbiol* **9**, 1581 (2018).
5. C. J. Pratt, E. E. Chandler, N. H. Youssef, M. S. Elshahed, T*estudinimyces gracilis* gen. nov, sp. nov. and *Astrotestudinimyces divisus* gen. nov, sp. nov., two novel, deep-branching anaerobic gut fungal genera from tortoise faeces. *Int J Syst Evol Microbiol* **73**, doi: 10.1099/ijsem.1090.005921. (2023).
6. R. A. Hanafy *et al.*, Phylogenomic analysis of the *Neocallimastigomycota*: proposal of *Caecomycetaceae* fam. nov., *Piromycetaceae* fam. nov., and emended description of the families *Neocallimastigaceae* and *Anaeromycetaceae*. *Int J Syst Evol Microbiol* **73** (2023).
7. Y. Wang *et al.*, Molecular dating of the emergence of anaerobic rumen fungi and the impact of laterally acquired genes. *mSystems* **4**, e00247-00219 (2019).

8. C. H. Haitjema *et al.*, A parts list for fungal cellulosomes revealed by comparative genomics. *Nat Microbiol* **2**, 17087 (2017).

9. J. L. Brown *et al.*, Co-cultivation of the anaerobic fungus *Caecomyces churrovis* with *Methanobacterium bryantii* enhances transcription of carbohydrate binding modules, dockerins, and pyruvate formate lyases on specific substrates. *Biotechnol Biofuels* **14**, 234 (2021).

10. J. K. Henske *et al.*, Transcriptomic characterization of *Caecomyces churrovis*: a novel, non-rhizoid-forming lignocellulolytic anaerobic fungus. *Biotechnol Biofuels* **10**, 305 (2017).

11. S. E. Wilken *et al.*, Experimentally validated reconstruction and analysis of a genome-scale metabolic model of an anaerobic *Neocallimastigomycota* fungus. *mSystems* **16**, e00002-00021 (2021).

12. Y. Li *et al.*, Combined genomic, transcriptomic, proteomic, and physiological characterization of the growth of *Pecoramyces* sp. F1 in monoculture and co-culture with a syntrophic methanogen. *Front Microbiol* **10**, 435 (2019).
