# Supplementary material for: Anaerobic fungi in the tortoise alimentary tract illuminate early stages of host-fungal symbiosis and Neocallimastigomycota evolution
Source: Nat Commun. 2024 Mar 28;15:2714. doi: 10.1038/s41467-024-47047-4 (PMC10978972; doi:10.1038/s41467-024-47047-4)
Supplement: Supplementary file 3 — Description of Additional Supplementary Files [file 41467_2024_47047_MOESM3_ESM.pdf]

Description of Additional Supplementary Files

File Name: Dataset S1
Description: AGF community composition in the studied tortoise samples.

File Name: Dataset S2
Description: AGF community structure of the samples from mammlian hosts used for comparative analysis. Samples shown in red text were used for AGF load quantification using qPCR.

File Name: Dataset S3
Description: Functional annotation of hydrogenosomal predicted peptides in one representative of each tortoise-associated AGF genus (strains B1.1 and T130A), as well as one representative of mammalian-associated AGF genera (Orpinomyces joyonii strain AB3). Green shaded cells indicate the function was detected in the hydrogenosomal predicted peptides from the transcriptome in the Table header.

File Name: Dataset S4
Description: CAZYme composition of the 52 mammalian-sourced AGF transcriptomes compared to the 7 tortoise-isolated AGF transcriptomes.

File Name: Dataset S5
Description: Mass spectrometry results. Peptides identified in mass spectrometry are shown for each strain along with the predicted function using KEGG, Conserved Domain Database, and dbcan4. Also shown are the intensities of each peptide in the biomass and cellulose bound fraction as well as the ratio of intensity in cellulose-bound fraction to the intensity in the biomass fraction.

File Name: Dataset S6
Description: Mass spectrometry intensities for the 221, and 96 proteins predicted to be cellulosomal from the transcriptomic analysis. The second column depicts whether the peptides were detected in the MS run. The "Predicted cellulosomal function" column indicates whether transcriptomics analysis predicted a non-catalytic dockerin domain (NCDD) or a scaffoldin function. CBM10 prediction using CDD database, other CDD domains, dbcan4 CAZy prediction, and Kegg predi ted function are shown for each peptide. Also shown are the intensities of each peptide in the biomass and cellulose-bound fraction as well as the ratio of intensity in cellulose-bound fraction to the intensity in the biomass fraction
