# Supplementary material for: Anaerobic fungi in the tortoise alimentary tract illuminate early stages of host-fungal symbiosis and Neocallimastigomycota evolution
Source: Nat Commun. 2024 Mar 28;15:2714. doi: 10.1038/s41467-024-47047-4 (PMC10978972; doi:10.1038/s41467-024-47047-4)
Supplement: Supplementary file 10 — Reporting Summary [file 41467_2024_47047_MOESM10_ESM.pdf]

nature portfolio

Corresponding author(s): Noha H. Youssef

Last updated by author(s): December 5, 2023

Reporting Summary

Nature Portfolio wishes to improve the reproducibility of the work that we publish. This form provides structure for consistency and transparency in reporting. For further information on Nature Portfolio policies, see our Editorial Policies and the Editorial Policy Checklist.

Statistics

For all statistical analyses, confirm that the following items are present in the figure legend, table legend, main text, or Methods section.

n/a	Confirmed	
☐	☒	The exact sample size (*n*) for each experimental group/condition, given as a discrete number and unit of measurement
☐	☒	A statement on whether measurements were taken from distinct samples or whether the same sample was measured repeatedly
☐	☒	The statistical test(s) used AND whether they are one- or two-sided<br>*Only common tests should be described solely by name; describe more complex techniques in the Methods section.*
☐	☒	A description of all covariates tested
☐	☒	A description of any assumptions or corrections, such as tests of normality and adjustment for multiple comparisons
☐	☒	A full description of the statistical parameters including central tendency (e.g. means) or other basic estimates (e.g. regression coefficient) AND variation (e.g. standard deviation) or associated estimates of uncertainty (e.g. confidence intervals)
☐	☒	For null hypothesis testing, the test statistic (e.g. *F*, *t*, *r*) with confidence intervals, effect sizes, degrees of freedom and *P* value noted<br>*Give P values as exact values whenever suitable.*
☒	☐	For Bayesian analysis, information on the choice of priors and Markov chain Monte Carlo settings
☒	☐	For hierarchical and complex designs, identification of the appropriate level for tests and full reporting of outcomes
☒	☐	Estimates of effect sizes (e.g. Cohen's *d*, Pearson's *r*), indicating how they were calculated

Our web collection on statistics for biologists contains articles on many of the points above.

Software and code

Policy information about availability of computer code

Data collection	For amplicon datasets: Read assembly, quality control to remove any sequence with average quality score <25, sequences with ambiguous bases, sequences not containing the correct barcode, sequences with more than 2bp difference in the primer sequence, and/or sequences with homopolymer stretches longer than 8bp was performed in Mothur (published software).<br>Transcriptomics data:RNA-seq reads were quality trimmed and de novo assembled using Trinity (v2.14.0) and default parameters (published software).<br>Proteomics data: RAW files from the mass spectrometer were searched against the corresponding transcriptome predicted peptides database using the MaxQuant application (v2.0.2.0 57). Searches utilized MaxQuant defaults, supplemented with two additional peptide modifications: deamidation of N/Q residues, and Q cyclization to pyroglutamate. The MaxQuant "match between runs" algorithm was not used. Sequences for reversed-sequence decoy proteins and common contaminants proteins were utilized for the database searches but were removed from the final MaxQuant protein results.
Data analysis	Code for phylogenomic analysis is available at https://github.com/stajichlab/PHYling_unified. Code used to create other figures is available at https://github.com/nohayoussef/AGF_Tortoises

For manuscripts utilizing custom algorithms or software that are central to the research but not yet described in published literature, software must be made available to editors and reviewers. We strongly encourage code deposition in a community repository (e.g. GitHub). See the Nature Portfolio guidelines for submitting code & software for further information.

Data

Policy information about availability of data

All manuscripts must include a data availability statement. This statement should provide the following information, where applicable:

- Accession codes, unique identifiers, or web links for publicly available datasets
- A description of any restrictions on data availability
- For clinical datasets or third party data, please ensure that the statement adheres to our policy

Illumina amplicon reads generated in this study have been deposited in GenBank under BioProject accession number PRJNA997953 (https://www.ncbi.nlm.nih.gov/bioproject/997953), and BioSample accession numbers SAMN36694530- SAMN36694536 (https://www.ncbi.nlm.nih.gov/biosample?LinkName=bioproject_biosample_all&from_uid=997953). RNA-seq reads from tortoise isolates have been deposited in GenBank under BioProject accession number PRJNA997953, and BioSample accession numbers SAMN36694608- SAMN36694614 (https://www.ncbi.nlm.nih.gov/biosample?LinkName=bioproject_biosample_all&from_uid=997953). Source data are provided with this paper.

Research involving human participants, their data, or biological material

Policy information about studies with human participants or human data. See also policy information about sex, gender (identity/presentation), and sexual orientation and race, ethnicity and racism.

Reporting on sex and gender	NA
Reporting on race, ethnicity, or other socially relevant groupings	NA
Population characteristics	NA
Recruitment	NA
Ethics oversight	NA

Note that full information on the approval of the study protocol must also be provided in the manuscript.

Field-specific reporting

Please select the one below that is the best fit for your research. If you are not sure, read the appropriate sections before making your selection.

☐ Life sciences ☐ Behavioural & social sciences ☒ Ecological, evolutionary & environmental sciences

For a reference copy of the document with all sections, see nature.com/documents/nr-reporting-summary-flat.pdf

Ecological, evolutionary & environmental sciences study design

All studies must disclose on these points even when the disclosure is negative.

Study description	Characterization of Anaerobic fungi in the tortoise alimentary tract
Research sample	A total of 11 fecal samples from 11 individual available tortoises were obtained
Sampling strategy	Since many of these tortoises are unaccessible in their natural habitats and many are endangered, sampling was conducted on animals housed in captivity (Oklahoma City Zoo and Hawk Hill farms, Oklahoma, USA). Only 11 total subjects were sampled due to availability
Data collection	Freshly deposited samples were placed in 15- or 50-mL sterile conical centrifuge tubes and transferred on ice to the laboratory, where they were stored at -20ºC. All samples originated from individual animals and were not adulterated during sampling with dust, dirt, or feces from other subjects. All sampled tortoises were visibly healthy during the time of sampling. Zoo personnell collected fecal samples.

Timing and spatial scale	Only one sample was collected per animal. 10 samples were obtained from OKC Zoo, and one was obtained from a local farm in Walters OK. Sampling occurred between November 2020 and March 2022
Data exclusions	No data was excluded from the analysis
Reproducibility	Reproducibility was assessed by comparing our results to previous studies.
Randomization	No randomization was used, given that this study is not set up like a clinical trial.
Blinding	No blinding was used, given that this study is not set up like a clinical trial.

Did the study involve field work? ☐ Yes ☒ No

Reporting for specific materials, systems and methods

We require information from authors about some types of materials, experimental systems and methods used in many studies. Here, indicate whether each material, system or method listed is relevant to your study. If you are not sure if a list item applies to your research, read the appropriate section before selecting a response.

Materials & experimental systems

n/a	Involved in the study
☒	☐ Antibodies
☒	☐ Eukaryotic cell lines
☒	☐ Palaeontology and archaeology
☒	☐ Animals and other organisms
☒	☐ Clinical data
☒	☐ Dual use research of concern
☒	☐ Plants

Methods

n/a	Involved in the study
☒	☐ ChIP-seq
☒	☐ Flow cytometry
☒	☐ MRI-based neuroimaging

Plants

Seed stocks	*Report on the source of all seed stocks or other plant material used. If applicable, state the seed stock centre and catalogue number. If plant specimens were collected from the field, describe the collection location, date and sampling procedures.*
Novel plant genotypes	*Describe the methods by which all novel plant genotypes were produced. This includes those generated by transgenic approaches, gene editing, chemical/radiation-based mutagenesis and hybridization. For transgenic lines, describe the transformation method, the number of independent lines analyzed and the generation upon which experiments were performed. For gene-edited lines, describe the editor used, the endogenous sequence targeted for editing, the targeting guide RNA sequence (if applicable) and how the editor was applied.*
Authentication	*Describe anya uthentication procedures for each seed stock used or novelgenotype generated. Describe anyexp eriments used to assess the effect of a mutation and, where applicable, how potential secondary effects (e.g. second site T-DNA insertions, mosiacism, off-target gene editing) were examined.*
